# Effect of Glucocorticosteroids in Diamond-Blackfan Anaemia: Maybe Not as Elusive as It Seems

**DOI:** 10.3390/ijms23031886

**Published:** 2022-02-08

**Authors:** Zuzana Macečková, Agáta Kubíčková, Juan Bautista De Sanctis, Marian Hajdúch

**Affiliations:** 1 Institute of Molecular and Translational Medicine, Palacky University Olomouc, Hnevotinska 1333/5, 77900 Olomouc, Czech Republic; zuzana.maceckova@upol.cz (Z.M.); agata.kubickova@upol.cz (A.K.); juabautista.desanctis@upol.cz (J.B.D.S.); 2 Czech Advanced Technology and Research Institute, Palacky University Olomouc, Hnevotinska 1333/5, 77900 Olomouc, Czech Republic

**Keywords:** Diamond-Blackfan anaemia, glucocorticosteroid, erythropoiesis, GATA1, c-myc, mTOR, autophagy

## Abstract

Diamond-Blackfan anaemia (DBA) is a red blood cell aplasia that in the majority of cases is associated with ribosomal protein (RP) aberrations. However, the mechanism by which this disorder leads to such a specific phenotype remains unclear. Even more elusive is the reason why non-specific agents such as glucocorticosteroids (GCs), also known as glucocorticoids, are an effective therapy for DBA. In this review, we (1) explore why GCs are successful in DBA treatment, (2) discuss the effect of GCs on erythropoiesis, and (3) summarise the GC impact on crucial pathways deregulated in DBA. Furthermore, we show that GCs do not regulate DBA erythropoiesis via a single mechanism but more likely via several interdependent pathways.

## 1. Introduction

Human erythrocytes differ from other cells because of their particular shape, lack of organelles, and transportation of oxygen via haemoglobin. Erythropoiesis is a complex differentiation process in which cells discard several organelles, from mitochondria to the nucleus, and dramatically increase haemoglobin production. These multifaceted changes need to be tightly regulated by numerous factors, and any disruption in signalling during erythropoiesis may lead to anaemia.

Diamond-Blackfan anaemia (DBA) [1] is a rare congenital disease usually diagnosed shortly after birth. Despite normal platelet and neutrophil counts, patients present with macrocytic or normocytic anaemia, reticulocytopenia, and a shortage of erythrocyte precursors [2]. Patients with DBA also manifest physical malformations, mainly craniofacial and upper limb, and growth retardation in approximately 35–50% of cases [3].

The first gene associated with DBA was RPS19 [4]. Interestingly, RPS19 variants account for approximately 25% of all DBA cases [5]. Over the last years, the number of RP genes associated with DBA increased; currently, there are 16 ribosomal protein aberrations linked to the disease, involving both large and small RP genes [6]. Rarely, non-ribosomal protein gene abnormalities were also associated with DBA, for instance in transcription factor GATA1 [7], iron metabolism gene *SLC49A1* [8], and ribosome maturation factor TSR2 [9]. The molecular pathology of approximately 20–50% of cases remains unknown; nonetheless, a number of candidate DBA genes is continually increasing with higher penetration of high-throughput DNA sequencing technologies into the clinic.

**Figure 1:**
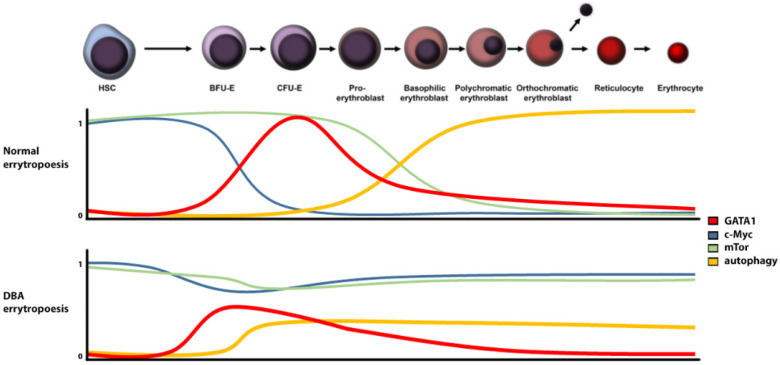
Regulation of relevant signalling proteins and pathways levels in DBA versus normal erythropoiesis (HSC: hematopoietic stem cells, BFU-E: burst-forming unit-erythroid, CFU-E: Colony-forming unit-erythroid).

The aberration of ribosomal proteins (RPs) results in a decreased number of available ribosomes with a consequent drop in translational activity [10]. A reduced number of ribosomes leads to an imbalance between translated and non-translated mRNA [11]. Furthermore, RP defects may result in ribosomal stress [12], p53 activation [12], altered mTOR signalling [13], c-Myc [14] deregulation, and other stress-related processes (Figure 1). Deregulation of any of these signal and metabolic pathways may result in anaemia.

As for many other rare diseases, therapeutic opportunities in DBA are inadequate. The first-line non-specific therapy usually includes glucocorticosteroids (GCs). However, treatment response is limited, and long-term use is associated with drug resistance and significant side effects [2]. Blood transfusion and iron chelation are usually reserved for GC non-responder [15]. Although some alternative therapies such as L-leucine supplementation to stimulate deficient protein synthesis have been used, the only permanent treatment of DBA is bone marrow transplantation [15]. However, bone marrow replacement is a high-risk procedure reserved for transfusion-dependent individuals. Recently, the approved gene therapy for betta thalassemia is also hope for many other congenital anaemias [16]. For DBA, gene therapy was already successfully tested in mice [17], and its further development is ongoing. Nevertheless, in contrast to thalassemia, where only hematopoietic cells are affected, DBA causes complex signalling and metabolic abnormalities in many other tissues, which will be difficult to correct by currently available gene therapy technologies.

Glucocorticosteroids (GCs) [18] generally improve erythropoiesis [19] and are frequently used for DBA treatment [3]. However, the mechanism by which GCs ameliorate DBA is elusive [19,20,21]. The present review aims to analyse the possible pathways that may be affected by GCs and improve DBA pathology. This is particularly important in the development of more efficient and targeted DBA therapies without glucocorticoid side effects.

## 2. Glucocorticosteroids

GCs are stress hormones produced in the adrenal cortex. They regulate diverse cellular functions, including development, homeostasis, metabolism, cognition, and inflammation [22]. GCs are widely used as treatments for a broad range of pathologies, from autoimmune syndromes and allergy to cancer [23]. Although GCs have a wide range of applications, their use is limited by severe adverse effects, especially in long-term or high-dose therapies. Possible side effects include osteoporosis, skin atrophy, diabetes, abdominal obesity, glaucoma, cataracts, avascular necrosis and infection, growth retardation, and hypertension [24].

Approximately 80% of DBA patients respond to initial GC treatment with a starting dose of 2 mg/kg per day for a maximum of 4 weeks to avoid GC toxicity in children [2,25]. The most serious GC adverse effects in infants include immunosuppression and susceptibility to infection, weight gain, growth retardation, osteonecrosis, and Cushingoid features [25].

The majority of GC effects are mediated by the glucocorticoid receptor (GCR). GCR is encoded by the *NR3C1* gene located on chromosome 5 and is a ligand-activated transcription factor. In an inactive form, GCR is predominantly located in the cytoplasm in complex with chaperone proteins and immunophilins [26]. However, its conformation changes upon ligand binding, whereupon it disassociates from the complex and translocates to the nucleolus, where it binds to GCR sequences and initiates the expression of target genes. Interestingly, GCR-mediated expression is tissue specific, and only a small number of genes are activated in all tissues [27]. This is probably caused by tissue-specific DNA methylation [28]. Furthermore, in addition to expression initiation, activated GCR can interact with other proteins. For example, it can modulate the activity of several kinases [29] and regulate GATA1 during erythropoiesis [30].

Moreover, GCR has several isoforms [31] and is highly polymorphic [32]. The genetic variability of GCR can affect body mass index, bone density or coronary artery diseases, probability of developing type II diabetes and GC treatment outcome [33,34]. This also applies in DBA. Surprisingly, the variability of GCR seems to have no or minor impact on GC treatment outcome in DBA [35]. However, it seems to modulate the onset of disease [35]. It has been shown that particular SNPs (rs6196 and rs860457) in GCR result in early onset of DBA, which can be caused by a modified GC response during embryogenesis [35].

## 3. The Primary Defect in DBA Cells

It is not fully understood how RP aberration causes red blood cell aplasia. A plausible mechanism involves a gap between transcription and translation [11]. The common feature of DBA is a decreased number of available ribosomes, resulting in decreased translational activity [10]. Several mRNAs are particularly affected by DBA ribosome impairment. Among the critically affected complexes are IRES-mediated mRNA and mRNAs with complex 5′ UTR regions [36]. 

GATA1, the specific erythropoiesis master regulator, is affected in DBA [37]. GATA1 is expressed during the final steps of erythrocyte development. It is responsible for triggering haemoglobin transcription, eliminating organelles and inducing other genes related to the terminal steps of erythropoiesis [38]. Although GATA1 mRNA levels are slightly increased or similar in RP depleted cells or DBA patients compared to controls, protein levels are downregulated [38]. The origin of this phenomenon is related to the complex 5′ UTR region of the gene [38]. GATA1 protein levels are restored and overall erythroid differentiation is improved when GATA1 5′ UTR region is substituted with less complex one [38]. 

It has been postulated that DBA red blood cells phenotype is induced by GATA1 translation inhibition [37]. GATA1 is expressed when committed cells are in the colony-forming unit-erythroid (CFU-E) stage of red blood cell (RBC) development [39]. At this stage, downregulation of GATA1 protein leads to apoptosis [40], which is consistent with the DBA phenotype. Furthermore, if RBC precursors escape apoptosis at the CFU-E stage, GATA1 decreased signalling may lead to impaired cell responses in later RBC developmental stages. Interestingly, mutations in the GATA1 gene have been described in several DBA-like patients [7,41]. However, although these patients showed impaired erythropoiesis similar to DBA, other typical features, such as physical malformations, were missing [7,41]. Furthermore, arsenic-induced disruption of GATA1 also leads to DBA-like erythropoiesis disruption [42].

Recent findings have suggested that erythropoiesis failure in DBA is more complicated than previously thought. More specifically, it seems that GATA1 impairment plays a crucial role in the DBA phenotype but only in ribosomal proteins of small-(RPS) and not large (RPL) subunit-related disorders [43].

## 4. GCs and GATA1

Impaired GATA1 protein levels seem to be the primary defect in DBA RBC precursor cells [44]. Therefore, it could be suggested that GCs restore GATA1 translation and related signalling pathways, and consequently increase erythrocyte precursor counts. However, GCs do not increase GATA1 but deplete it. GCs inhibit GATA1 by transcriptional repression and direct interaction of GCRs with GATA1 [25]. GCs also decrease protein synthesis in cells through mTOR and subsequent S6 kinase inhibition [45]. This reduction affects the levels of HSP70 [46], a chaperone that protects GATA1 from caspase-3 degradation during erythropoiesis [40]. Therefore, GCs do not seem to increase GATA1 levels but instead deplete them.

GCs can promote erythropoiesis by two mechanisms to overcome GATA1 impairment. Firstly, GCs can act before the CFU-E stage and increase the number of CFU-E precursors. Secondly, even though GCs do not rescue GATA1, they can regulate GATA1-related steps in erythropoiesis, as discussed further below.

## 5. Role of GCs in CFU-E Precursors

**Figure 2:**
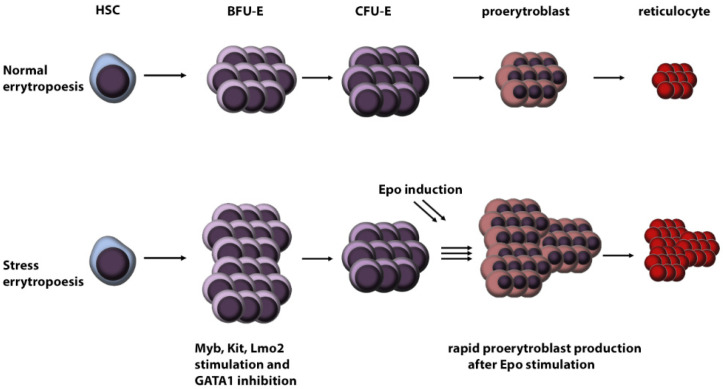
Schematic overview of stress versus normal erythropoiesis. (HSC: hematopoietic stem cells, BFU-E: burst-forming unit-erythroid, CFU-E: colony-forming unit-erythroid, Epo: erythropoietin).

Stress erythropoiesis may be caused by blood loss, oxygen deprivation, haemolytic anaemia and long-term stress [47] (Figure 2). This type of erythropoiesis leads to the rapid proliferation of RBC precursors driven by erythropoietin (Epo) and GCs [21]. Because DBA fulfils several criteria, we can hypothesise that stress erythropoiesis plays a crucial role in RBC development in DBA patients. 

When the body is under stress, cortisol, the intrinsic master GC, induces many physiological responses. One of them is to stimulate the production of Epo [48] in the kidneys [49]. Epo in turn stimulates CFU-E, and its primary target is GATA1 [50]. Due to GATA1 impairment, DBA is one of very few Epo insensitive anaemias [51]. Therefore, we can speculate that Epo induction via stress erythropoiesis probably does not lead to DBA phenotype improvement. 

GCs induce stress erythropoiesis also directly by activating GCR [19]. Furthermore, activation of GCR is conditional for stress erythropoiesis [19] and stimulates several transcription factors necessary for burst forming unit-erythroid (BFU-E), CFU-E precursor, cell proliferation, specifically Myb, Kit and Lmo2 [52]. Together with GATA1 inhibition, these transcription factors stimulate the rapid proliferation of BFU-E cells. This induction pathway is specific to BFU-E cells and increases the number of CFU-E precursor cells by up to 10-fold [53]. Thus, an increased number of CFU-E cells are generated, which, after Epo stimulation, undergo rapid differentiation [47]. Hence, stress erythropoiesis results in a significant increase in RBC number originating from one precursor cell.

Furthermore, it seems that stress erythropoiesis impairment plays a crucial role mainly in RPL-related DBA, and its external stimulation by GC treatment may result in restoration of this pathway [43].

## 6. Regulation of Ribosomal Stress and p53

It has been suggested that apoptosis in DBA RBC precursors is caused by p53 induction via ribosomal stress [54]. However, this hypothesis has been partially refuted since the haploinsufficiency of most RPs does not lead to p53 stimulation via ribosomal stress mechanisms [55]. Even though ribosomal stress and p53 signalling are critical for normal erythropoiesis [56], the activation of p53 in DBA is most likely mediated by impaired metabolism and DNA damage [6,57]. 

Unfortunately, it is difficult to elucidate the interplay between p53 and GCs. On the one hand, GCs decrease p53 levels [58], whereas on the other hand, increased p53, common in DBA cells, limits proper GCs receptor activation [59]. Moreover, GCs do not only regulate p53 directly but also indirectly by reducing p53 activation signals. 

As mentioned above, in addition to ribosomal stress, p53 could be activated by HEM-mediated ROS production [60,61]. Although it has been reported that GCs reduce ROS species [62,63,64], their overall influence on ROS production is ambiguous [65]. More specifically, it seems that short-term GC usage leads to a reduction in ROS generation, whereas long-term exposure leads to ROS induction [65]. Furthermore, increased ROS levels have been detected in DBA patients regardless of treatment [60]. Therefore, GC-mediated p53 regulation does not seem to reflect ROS levels. Thus, the precise mechanism by which GCs modulate p53 levels in DBA remains mysterious. Nevertheless, we can conclude that GC treatment improves p53 status in DBA [66].

## 7. Regulation of Enucleation through c-Myc

Enucleation is a critical step in erythropoiesis, in which the cells lose a major part of the nucleus to generate reticulocytes. Inhibition of this process may explain reticulocytopenia in DBA patients [67]. Before enucleation, the nucleolus must be condensed and histones deacetylated. 

Protein c-Myc is a master transcription factor that promotes and regulates proliferation, transcription, translation and cellular metabolism with an important role in several cell transduction pathways [68]. GATA1 silences c-Myc expression in RBC precursors [37], which highlights the importance of c-Myc during erythropoiesis. Only a slight increase in c-Myc levels disables enucleation and reticulocyte development by impairing histone deacetylases [68]. Activated c-Myc also induces proerythroblast apoptosis during RBC development [68]. 

c-Myc is deregulated in DBA in multiple ways. Downregulated GATA1 is probably the central factor leading to upregulation of c-Myc in all DBA erythroid progenitors. Interestingly, some RPs affected in DBA regulate c-Myc on their own, e.g., RPL11 and RPL5. Both these RPs regulate ribosomal stress induction [69] and c-Myc regulation [70] at mRNA and protein levels [14]. Impairment of RPL5 and RPL11 has also been associated with a more severe DBA phenotype [71]. In addition, c-Myc driven lymphomagenesis has been observed in a DBA mouse model [14]. Interestingly, similar or identical mutations of those RPs reported in DBA have also been detected in some cancers [72,73], characterized by p53 deactivation and activation of c-Myc signalling.

GCs transcriptionally repress c-Myc expression [74], leading to cell cycle arrest without apoptosis, which is necessary for proper RBC differentiation [75]. In DBA, GCs may support enucleation during erythropoiesis, and thus prevent reticulocytopenia by downregulating c-Myc.

## 8. mTOR Pathway and Autophagy

Autophagy is crucial in RBC development [76]. During erythrocyte differentiation, cells discard and/or recycle most subcellular structures, starting from mitochondria to the nucleus. The nucleus is removed through the enucleation step, whereas other organelles are typically removed via autophagy [77]. Autophagy is activated by GATA1 [78] and mTOR [79] mediated induction of autophagy genes transcription. Notably, increased mTOR leads to macrocytic anaemia [80], consistent with the DBA phenotype [81].

mTOR has a dual role in RBC development [82]. Its activity is necessary for proper BFU-E and CFU-E cell proliferation in the primary steps of RBC development. But in later steps of erythropoiesis, it needs to be silenced for autophagy induction. mTOR is known to be activated in DBA cells [13]. Activated mTOR in DBA probably facilitates the increased proliferation of BFU-E and CFU-E cells and in a back-loop manner stimulates deficient protein translation S6-kinase activation and in turn RPS6 phosphorylation [83].

Furthermore, L-leucine, an inductor of mTOR activation, is routinely used as a DBA treatment but with a variable response rate [84,85]. Thus, we suggest induction of mTOR is beneficial for DBA cells, especially during RBC development until the CFU-E stage. On the other hand, constitutive mTOR induction during terminal differentiation may lead to autophagy failure, followed by aberration of the final erythropoiesis steps. We can speculate that this may be the underlying mechanism in DBA because DBA erythroid precursors treated by rapamycin, an mTOR inhibitor, have been shown to increase the proliferation of late erythroid cells in DBA [86].

In DBA, autophagy is increased despite mTOR activation [13]. It seems to be induced by ROS [60], hypoxia [87] or some mTOR-independent pathways [88]. However, mTOR-independent autophagy may not be sufficient for proper RBC development [89]. Therefore, autophagy induction may offer a possible target for future treatment of DBA patients [86].

**Figure 3:**
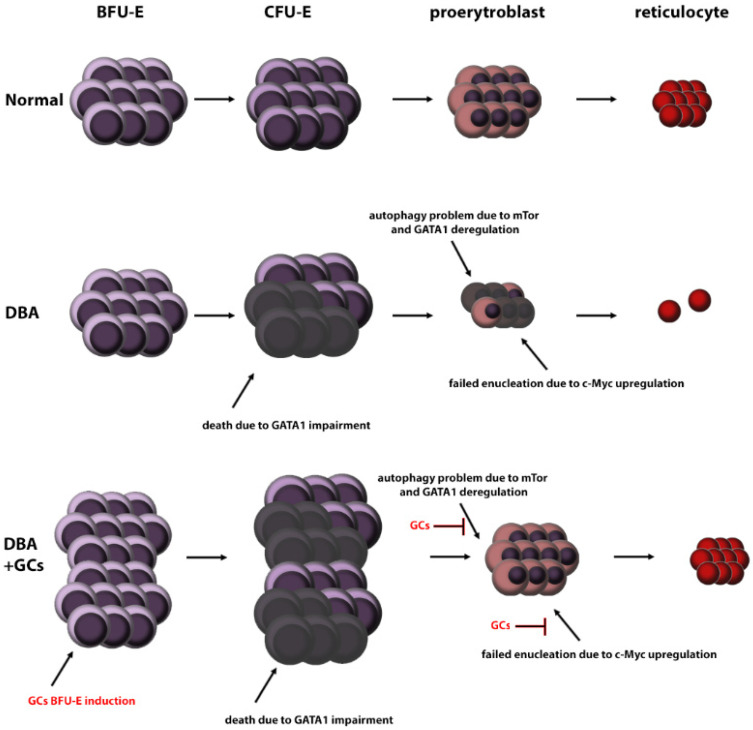
Possible mechanism involved in GC-mediated improvement of DBA erythropoiesis. (HSC: hematopoietic stem cells, BFU-E: burst-forming unit-erythroid, CFU-E: colony-forming unit-erythroid, DBA: Diamond Blackfan anemia, GC: glucocorticosteroids).

Inhibition of mTOR by GCs followed by autophagy has been described in several cell types [90,91,92]. Therefore, a plausible hypothesis is that GCs may also improve erythropoiesis by modulating mTOR, and subsequently autophagy. The other important pathway is inactivation of Akt signalling by GCs in DBA cells [13,93]. Even though GC-induced mTOR inactivation may lead to decreased proliferation of BFU-E and CFU-E cells [82], stress-induced erythropoiesis may compensate for this [39] (Figure 3).

## 9. GC Resistance

GC resistance is common in almost all GC-based therapies [94,95]; DBA is no exception [96]. Multiple mechanisms may induce GC resistance, and these mechanisms may vary among individual patients [97]. GCR polymorphism, alternative splicing and downregulation of GCR expression may also lead to GCR resistance [97], expression of pharmacological multidrug transporters may also be involved [94]. GC resistance in DBA has been shown to be associated with p57 cyclin dependent kinase inhibitor dysregulation [96]. Expression of p57 is directly induced by the GCR responsive element promoter [98]. Therefore, more than one mechanism may play a role to effect GC resistance.

## 10. Conclusions

GCs have been used to treat DBA for decades, but their mechanism of action remains elusive, and it is difficult to understand them fully due to their pleiotropic effects. We suggest that GCs improve the DBA pathology via at least four relatively independent ways: (i) induction of BFU-E cell proliferation via stress erythropoiesis, (ii) (de)regulation of P53 signalling, (iii) deactivation of c-Myc, and (iv) inhibition of mTOR signalling. Altogether, these four steps result in the induction of BFU-E cell proliferation and terminal differentiation to RBCs.

Although all four pathways seem to be relevant in DBA treatment, to date, only L-leucine modulation of mTOR activity is routinely used as a therapy [84]. Stress erythropoiesis seems to be tightly connected with GCs as stress hormones [19], and there is currently no evidence for other biologically active compounds. Protein p53 [99] and c-Myc [100] inhibitors are tested in cancer clinical trials, but their efficacy in DBA patients or DBA preclinical models is unknown. Thus, further studies are needed to ascertain the role of the above-mentioned GC-dependent pathways in the regulation and pathology of DBA, with the ultimate goal of developing new, more effective, and less toxic therapies for children suffering from this disease.
